# Supplementary material for: Clonal analysis reveals differential PGC contributions to the early germline and ovarian reserve
Source: bioRxiv. 2025 May 7:2025.04.30.651497. Preprint. [Version 1] doi: 10.1101/2025.04.30.651497 (PMC12247702; doi:10.1101/2025.04.30.651497)
Supplement: Supplement 1 [file media-1.pdf]

Clonal analysis reveals differential PGC contributions to the early germline and ovarian reserve

Maya Pahima[1] and Florence L. Marlow[1]*

[1]Department of Cell, Development, and Regenerative Biology – the Icahn School of Medicine at Mount Sinai, New York, NY, USA.

*Corresponding author. Email: florence.marlow@mssm.edu

Supplementary Materials

Supplementary Fig. 1: GSC fate location.

(A) Schematic of a d16 gonad (teal, volumetric) with *nos2*+ (yellow) clusters within to represent positioning of clusters. (**B)** Scatter plot showing the number of *nos2*+ cell clusters found per d16 gonad (n=23 gonads examined, n=56 *nos2*+ clusters, mean: 2.45, indicated in red with SD bar). (**C)** Bar graph comparing the positions of *nos2*+ clusters within gonads (periphery n=28, inside n=12, isolated n=16)

Supplementary Fig. 2: Germ traCre clonal analysis of samples with only *foxl2l* labeling.

(A) Bar graph showing clone type ratios of 195 analyzed clones (*nos2*+: 17, 8.72%; *foxl2l*+: 63, 32.31%; *nos2*+/*foxl2l*+: 4, 2.05%; *nos2*-/*foxl2l*-: 50, 25.64%; *foxl2l*-: 61, 31.28%). **(B)** Bar graph showing the distribution ratios of clone types along the gonadal AP axis. To quantify this, gonads separated into 4 quadrants (Q1-Q4) for analysis. Total number of clones per quadrant labeled. **(C)** Scatter plot of clone size distributions of 217 clones along the gonadal AP axis. Means indicated in red with black SD bars (Q1: 46, 4.85 ± 4.93; Q2: 46, 5.00 ± 6.18; Q3: 78, 3.78 ± 6.54; Q4: 67, 4.27 ± 6.39). **(D)** Scatter bar plot of clone sizes between clone types, SD bars indicated (*nos2*+: 17, 2.18 ± 1.19; *foxl2l*+: 28, 3.68 ± 3.32; *nos2*+/*foxl2l*+: 4, 2.50 ± 0.58; *nos2*-/*foxl2l*-: 50, 1.92 ± 1.58; *foxl2l*-: 61, 4.51 ± 6.57). **(E)** Bar plot showing ratio of one-cell clones to multi-cell clones per clone type (*nos2*+: 7:10, 41.18%; *foxl2l*+: 15:48, 23.81%; *nos2*+/*foxl2l*+: 0:4, 0%; *nos2*-/*foxl2l*-: 32:18, 64%; *foxl2l*-: 24:37, 39.34%).

Supplementary Figure 3: Number of colors observed in Germbow adult mosaic testes.

(A) Germbow mosaic testes. Scale bar: 500 μm. **(B)** Scatter plot showing the number of unique colors identified across wholemount mosaic Germbow testes (n=6). Mean indicated in red with black SD bars (4.33 ± 2.73).

Supplementary Figure 4: Germbow egg color lineage tracing bar graph time course representations.

Bar graphs representing egg color ratios over mating assay time course for **(A)** females with Cre low recombination and **(B-E)** females with high recombination.

Supplementary Table 1: Cloning reagents and sources for the Germ traCre, Germbow, and pACre-nos3 constructs.

Supplementary Table 2: Antibodies and probes for fluorescent staining.

Supplementary Data: Spreadsheet of mosaic Germbow female egg data.

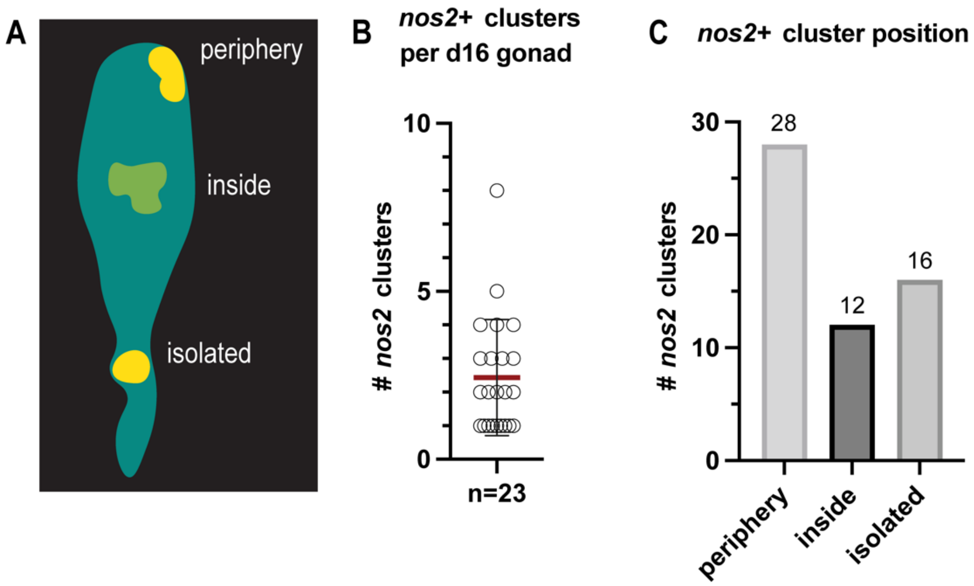

Supplementary Fig. 1: GSC fate location. (A) Schematic of a d16 gonad (teal, volumetric) with *nos2*+ (yellow) clusters within to represent positioning of clusters. (**B)** Scatter plot showing the number of *nos2*+ cell clusters found per d16 gonad (n=23 gonads examined, n=56 *nos2*+ clusters, mean: 2.45, indicated in red with SD bar). (**C)** Bar graph comparing the positions of *nos2*+ clusters within gonads (periphery n=28, inside n=12, isolated n=16)

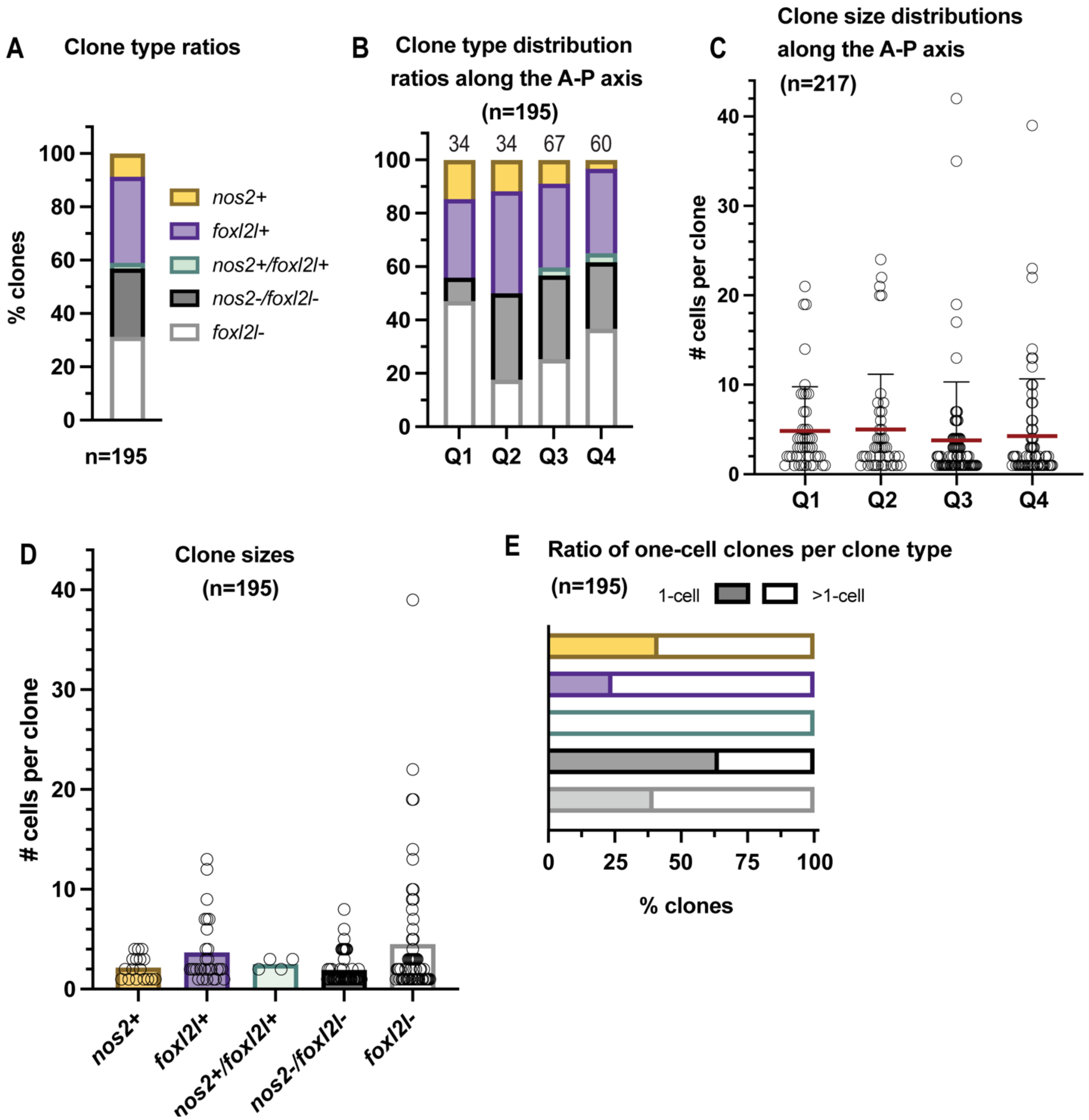

Supplementary Fig. 2: Germ traCre clonal analysis of samples with only *foxl2l* labeling. (A) Bar graph showing clone type ratios of 195 analyzed clones (*nos2*+: 17, 8.72%; *foxl2l*+: 63, 32.31%; *nos2*+/*foxl2l*+: 4, 2.05%; *nos2*-/*foxl2l*-: 50, 25.64%; *foxl2l*-: 61, 31.28%). **(B)** Bar graph showing the distribution ratios of clone types along the gonadal AP axis. To quantify this, gonads separated into 4 quadrants (Q1-Q4) for analysis. Total number of clones per quadrant labeled. **(C)** Scatter plot of clone size distributions of 217 clones along the gonadal AP axis. Means indicated in red with black SD bars (Q1: 46, 4.85 ± 4.93; Q2: 46, 5.00 ± 6.18; Q3: 78, 3.78 ± 6.54; Q4: 67, 4.27 ± 6.39). **(D)** Scatter bar plot of clone sizes between clone types, SD bars indicated (*nos2*+: 17, 2.18 ± 1.19; *foxl2l*+: 28, 3.68 ± 3.32; *nos2*+/*foxl2l*+: 4, 2.50 ± 0.58; *nos2*-/*foxl2l*-: 50, 1.92 ± 1.58; *foxl2l*-: 61, 4.51 ± 6.57). **(E)** Bar plot showing ratio of one-cell clones to multi-cell clones per clone type (*nos2*+: 7:10, 41.18%; *foxl2l*+: 15:48, 23.81%; *nos2*+/*foxl2l*+: 0:4, 0%; *nos2*-/*foxl2l*-: 32:18, 64%; *foxl2l*-: 24:37, 39.34%).

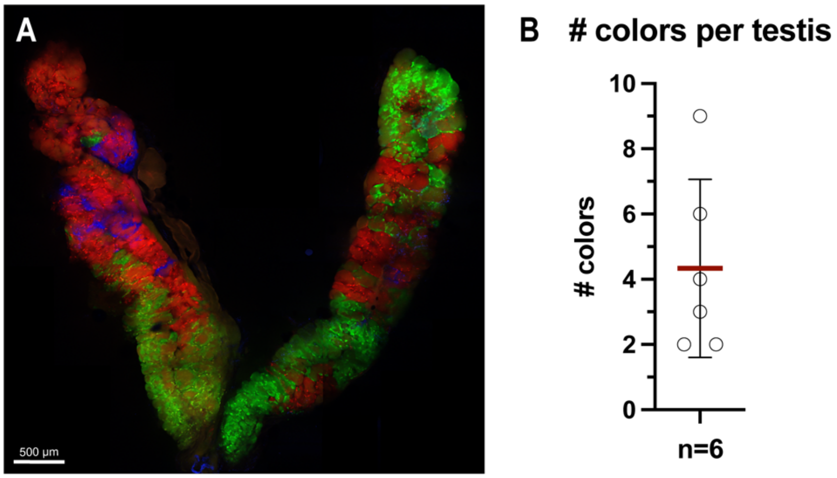

Supplementary Figure 3: Number of colors observed in Germbow adult mosaic testes. (A) Germbow mosaic testes. Scale bar: 500 µm. **(B)** Scatter plot showing the number of unique colors identified across wholemount mosaic Germbow testes (n=6). Mean indicated in red with black SD bars (4.33 ± 2.73).

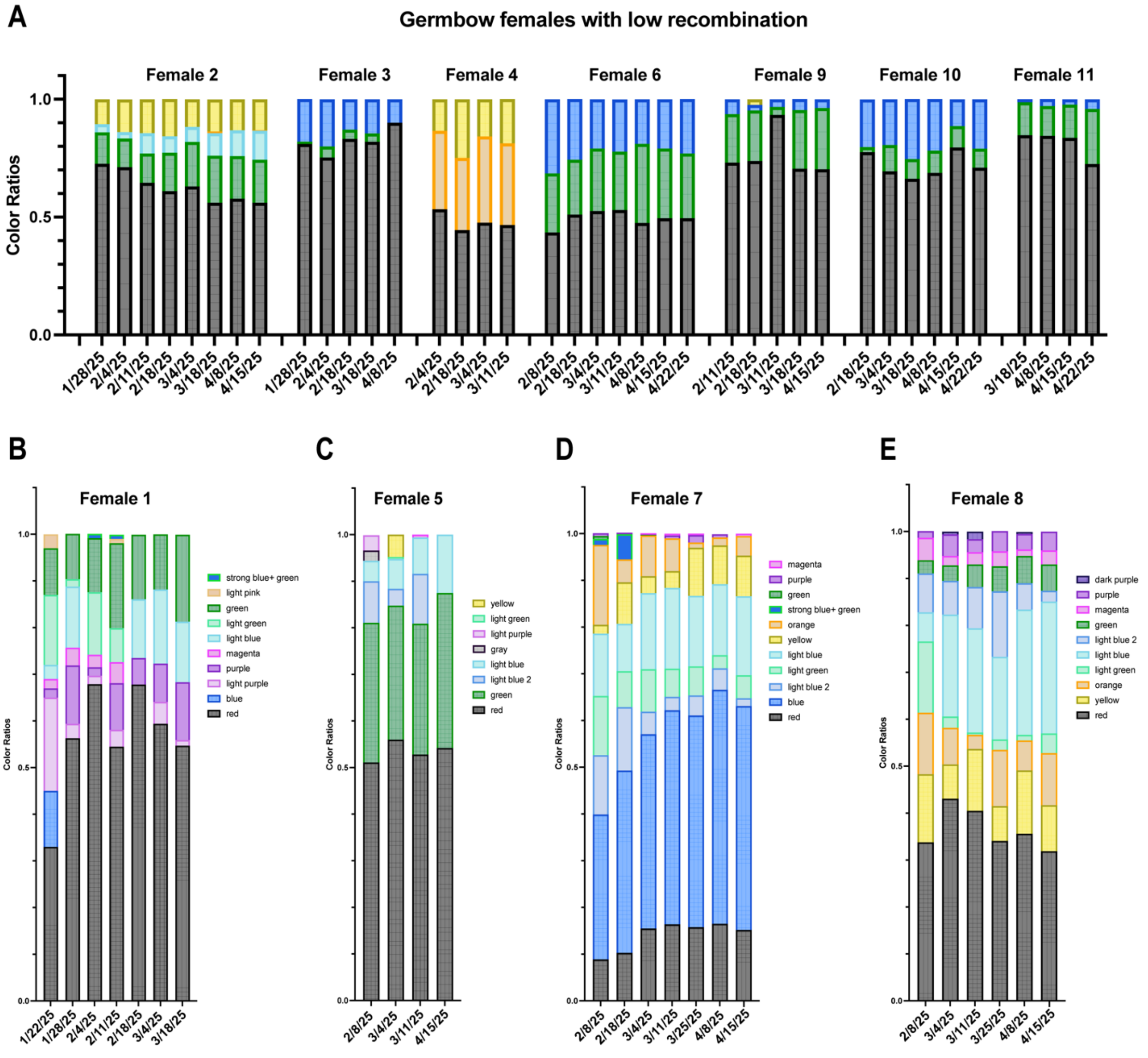

Supplementary Figure 4: Germbow egg color lineage tracing bar graph time course representations. Bar graphs representing egg color ratios over mating assay time course for **(A)** females with Cre low recombination and **(B-E)** females with high recombination.

Supplementary Table 1

Plasmids +A2:E24used	Plasmids used	Plasmids used	Plasmids used	Plasmids used
pCS2FA-transposase	Tol2kit v1.0	#396	(Kwan *et al.* , 2007)	
pME-iCre (improved Cre)	Tol2kit v2.0	#589	(Sztal *et al.* , 2015)	
pTol2-ziwi:loxp-stop-loxp-mcherry; cmlc2:gfp	generated in this study, Addgene	#226785		*ziwi:stop-mCherry, pGH* (**Germ traCre**)
pTol2-R4/R3_cmlc2:gfp	Tol2kit v1.0	#393	(Kwan *et al.* , 2007)	*pDestTol2CG*
p5E-ziwi	gift from Dr. Bruce Draper		(Leu and Draper, 2010)	
pME-loxp-stop-loxp	Tol2kit v2.0	#727		
p3E-mcherry	Addgene, Dr. Rob Parton	#108884	(Ariotti *et al.* , 2018)	
pCSDest2-pACre-nos3,3'utr	generated in this study, Addgene	#226790		*p+E12BH-R4/R2+A2:E24*
pCSDest2	Addgene, Dr. Nathan Lawson	#22424	(Villefranc et al., 2007)	
p3E-nos3,3'utr	Gift from Dr. Erez Raz		(Blaser et al., 2005)	
pME-pACre	generated in this study, Addgene	#226789		
pME-MCS (Multi Cloning Site)	Tol2kit v2.0			
pcDNA3.1-pACre	Addgene, Dr. Moritoshi Sato	#122960	(Kawano et al., 2016)	
pTol2-ziwi:Zebrabow; cmlc2:mCherry	generated in this study, Addgene	#226787		*ziwi:Zebrabow, pBH* (**Germbow**)
pTol2Dest-R4/R2_cmlc2:mCherry	Marlow lab		(Heim et al., 2014)	*pBH-R4/R2*
p5E-ziwi	gift from Dr. Bruce Draper		(Leu and Draper, 2010)	
pME-Zebrabow	generated in this study, Addgene	#226792		
Zebrabow_GateDest	Addgene, Dr. Albert Pan	#118221	(Pan et al., 2013)	
pME-MCS (Multi Cloning Site)	Tol2kit v2.0			

Supplementary Table 2

Primary antibodies and probes	Dilution/Conc.	Source
Rabbit anti-Ddx4/Vasa (polyclonal)	1:2000	PMID:10811828
Chicken anti-Ddx4/Vasa (polyclonal)	1:2000	PMID: 30653507
nanos2	16nM	Molecular Instruments (PRB905)
foxl2l	8nM	Molecular Instruments (PRD331)
rec8a	16nM	Molecular Instruments (PRE326)
bicc1a	16nM	Molecular Instruments (RTP763)
